# Complex regional pain syndrome type II caused by iatrogenic lateral dorsal cutaneous nerve injury: A case report

**DOI:** 10.1097/MD.0000000000028108

**Published:** 2021-12-10

**Authors:** Tae-Hoon Kim, Geun-Yeol Jo, Wanil Kim, Hwan-Kwon Do

**Affiliations:** a Department of Physical Medicine and Rehabilitation, Haeundae Paik Hospital, Inje University College of Medicine, Busan, Republic of Korea; b Department of Biochemistry, Department of Convergence Medical Science, and Institute of Health Sciences, Gyeongsang National University College of Medicine, Jinju, Republic of Korea.

**Keywords:** complex regional pain syndrome, lateral dorsal cutaneous nerve, paresthesia, peripheral nerve injury, sural nerve

## Abstract

**Rationale:**

Complex regional pain syndrome (CRPS) is a painful condition classified as type I or II depending on the absence or presence of nerve injury, respectively. Injury to the lateral dorsal cutaneous nerve (LDCN), a branch of the sural nerve, is a rare occurrence observed after a sprain or procedures conducted on the lateral side of the ankle.

**Patient concerns:**

A 38-year-old female, who had undergone prolotherapy for a sprain in the lateral side of the left ankle 3 months ago, presented with persistent causalgia and dysesthesia around the injection site.

**Diagnosis:**

An electrodiagnostic study was conducted, which confirmed that the patient had peripheral neuropathy of the left LDCN. Considering the digital infrared thermal imaging and three-phase bone scan findings and the clinical presentation, the condition was diagnosed as CRPS type II due to iatrogenic LDCN injury according to the Budapest diagnostic criteria for CRPS.

**Interventions:**

The patient was treated with steroid pulse therapy, physical therapy, and transcutaneous electrical nerve stimulation, as well as nonsteroidal anti-inflammatory drugs, pregabalin, and tricyclic antidepressants.

**Outcomes:**

After 1 month of treatment, allodynia of the left foot persisted, but the pain reduced from 6 points to 3 points on the numeric rating scale. Partial recovery of amplitude and conduction velocity was confirmed in the follow-up electrodiagnostic study.

**Lessons:**

LDCN injury should be considered in patients who complain of persistent lateral ankle and foot paresthesia or pain after sprain or procedures performed on the lateral side of the ankle. Early diagnosis and treatment can lead to a good prognosis when the LDCN injury has progressed to CRPS.

## 1 Introduction

Complex regional pain syndrome (CRPS) encompasses a wide range of pain conditions, including both acute and chronic pain conditions. It is characterized by pain that is irregular in intensity or duration relative to the general pain process and spontaneous or induced local pain originating from the distal parts of the extremities.^[1,2]^

Generally, CRPS is caused by physical injuries such as fractures, sprains, or surgeries. The pathogenesis of CRPS is reportedly due to abnormal inflammatory processes, including hypoxia, reperfusion injury, and the release of pro-inflammatory agents. Additionally, studies have reported that CRPS is affected by the dysregulation of the autonomic nervous system and central nervous system, autoimmune disorders, small fiber neuropathy, and genetic factors.^[1,3]^

CRPS is classified as type I or type II according to the presence of proven neurological lesions. CRPS type I presents without nerve damage and is known to be more common than CRPS type II.^[2–4]^

The sural nerve (SN) is one of the main sensory nerves of the lower extremities. It is responsible for the cutaneous sensations in the posterior and lateral aspects of the legs, lateral calcaneal area of the distal third of the legs, and lateral aspect of the feet and fifth toes.^[5,6]^ The SN originates from medial and lateral cutaneous nerves branching from each of the tibial and common peroneal nerves, joining just distal of the popliteal fossa. It then descends between the two heads of the gastrocnemius muscle and passes through the lateral margin of the calcaneal tendon and between the lateral malleolus and calcaneus. After passing under the lateral malleolus, it leads toward the lateral dorsal cutaneous nerve (LDCN), which is responsible for the sensation of the dorsolateral aspects of the foot and terminates in the lateral aspects of the fifth toes of the feet.^[5–7]^

LDCN injury is a rare injury that can occur during a lateral ankle sprain or as an iatrogenic injury during surgery of the Achilles tendon or lateral side of the ankle. This injury can lead to pain, loss of sensation, and painful neuroma.^[6–8]^ Although several studies have reported unexplained postoperative pain and paresthesia of the lateral aspects of the feet after foot surgeries for fifth metatarsal fractures, complications resulting from LDCN injury, a branch of the SN, have remained underreported and largely unrecognized.^[9]^

When iatrogenic nerve injury occurs, early diagnosis and planning for further management can positively affect the outcomes. Furthermore, active treatment in the early stage of CRPS improves its prognosis.^[10,11]^ This case report is the first to describe the experience of using a diagnostic approach and treatment in a patient with iatrogenic LDCN injury who progressed to CRPS.

## 2 Case presentation

### 2.1 Patient information

A 38-year-old female patient reported experiencing a sharp pain while receiving prolotherapy for her sprained left ankle at a local clinic 3 months ago. The burning pain and abnormal sensation in her left ankle remained persistent since then, for which she visited our hospital. She was a homemaker, and there was no abnormal pain in the ankle before the sprain. After the prolotherapy, the abnormal pain gradually worsened over time.

### 2.2 Clinical findings

Physical examination revealed that the patient had numbness along with a tingling sensation on the lateral aspect of the left ankle and foot. She had hypersensitivity, allodynia, and causalgia, and the pain score was 6 on the numeric rating scale. Skin swelling and redness were observed in the affected area, and the pain spread to the distal part of the leg. In manual muscle testing, the ankle strength, including ankle plantarflexion and dorsiflexion, was at grade 5, which was considered normal. Nevertheless, the patient complained of subjective weakness and painful range of motion (ROM) movement, but the passive ROM of the ankle joint was normal.

### 2.3 Diagnostic assessment

**Figure 1:**
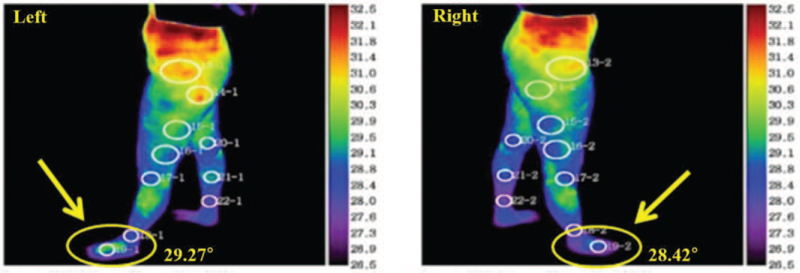
Digital infrared thermal imaging showed higher temperature in the left (affected) foot than in the right (unaffected) foot.

**Figure 2:**
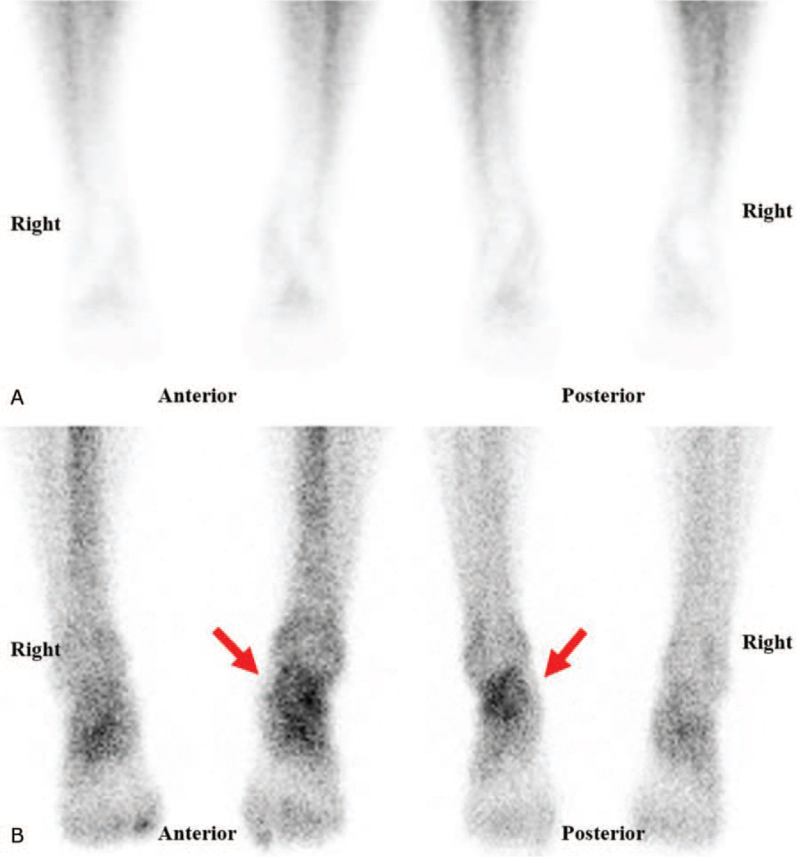
Three-phase bone scan showed increased uptake in the left ankle region in the osseous phase. (A) Blood flow and pool phase; (B) osseous phase.

We performed an electrodiagnostic examination using Viking Select (Nicolet, San Carlos, CA) to check for nerve damage. In the motor nerve conduction study of the left peroneal nerve and tibial nerve, the latency and amplitude were within the normal range. However, in the sensory nerve conduction study of the left LDCN, the amplitude and conduction velocity were decreased and the onset latency was delayed compared to the contralateral side (Table 1). No denervation potential was observed on needle electromyography of the left lower extremity. Based on the electrodiagnostic findings, the condition was diagnosed as peripheral neuropathy of the left LDCN of the SN. Following this, the magnetic resonance imaging of the left ankle was performed to check for any anatomical abnormalities of the ankle, neural compression, and soft tissue problems, but no specific abnormalities were found. In the digital infrared thermal imaging (Medicore, Seongnam, Gyeonggi, Korea) performed to check temperature asymmetry, the temperature of the affected left side was 0.85° higher than that of the contralateral side, confirming vasomotor abnormality (Fig. 1). A three-phase bone scan using technetium-99 m methyl diphosphonate showed symmetric blood flow to both lower extremities and feet in the blood flow and pool phase, but increased uptake in the left ankle was confirmed in the delayed static bone scan (Osseous phase) (Fig. 2).

**Table 1:** Results of nerve conduction study.

Nerve	Stimulation site	Latency (ms)	Amplitude (mV)	NCV (m/s)
Motor				
Lt peroneal (EDB recording)	Ankle	3.3	5.8	
	Fibula head	9.5	5.7	51.2
	Popliteal fossa	10.8	5.6	55.1
Lt tibial (AH recording)	Ankle	3.0	24.4	
	Popliteal fossa	10.6	16.0	51.4
Sensory				
Lt sural	Calf	3.5	22.1	40.5
Rt sural	Calf	3.9	27.6	35.7
Lt LDCN	Ankle	3.3	7.6	30.8
Rt LDCN	Ankle	3.0	14.9	36.7
Lt superficial peroneal	Calf	3.3	11.9	42.5

Considering the results of these tests and the patient's clinical features, we concluded that the findings fulfilled the Budapest diagnostic criteria for CRPS; hence, a diagnosis of CRPS type II due to iatrogenic LDCN injury was made (Table 2).

**Table 2:** Budapest clinical diagnostic criteria for CRPS.

**1**	Continuing pain, which is disproportionate to any inciting event
**2**	Must report at least one symptom in *three of the four* following categories:
	•* Sensory:* reports of hyperesthesia and/or allodynia
	•* Vasomotor:* reports of temperature asymmetry and/or skin color changes and/or skin color asymmetry
	•* Sudomotor/edema:* reports of edema and/or sweating changes and/or sweating asymmetry
	•* Motor/trophic:* reports of decreased range of motion and/or motor dysfunction (weakness, tremor, dystonia) and/or trophic changes (hair, nail, skin)
**3**	Must display at least one sign at time of evaluation in *two or more* of the following categories:
	•* Sensory:* evidence of hyperalgesia (to pinprick) and/or allodynia (to light touch and/or deep somatic pressure and/or joint movement)
	•* Vasomotor:* evidence of temperature asymmetry and/or skin color changes and/or asymmetry
	•* Sudomotor/edema:* evidence of edema and/or sweating changes and/or sweating asymmetry
	•* Motor/trophic:* evidence of decreased range of motion and/or motor dysfunction (weakness, tremor, dystonia) and/or trophic changes (hair, nail, skin)
**4**	There is no other diagnosis that better explains the signs and symptoms

### 2.4 Therapeutic intervention

The patient received steroid pulse therapy at a titrating dose of 40 mg of prednisolone for 2 weeks, followed by physical therapies including desensitization, isometric exercises of the ankle, resisted ROM, and transcutaneous electrical nerve stimulation for 4 weeks. Additionally, non-steroidal anti-inflammatory drugs, pregabalin, and tricyclic antidepressants were prescribed for analgesia.

### 2.5 Follow-up and outcomes

**Figure 3:**
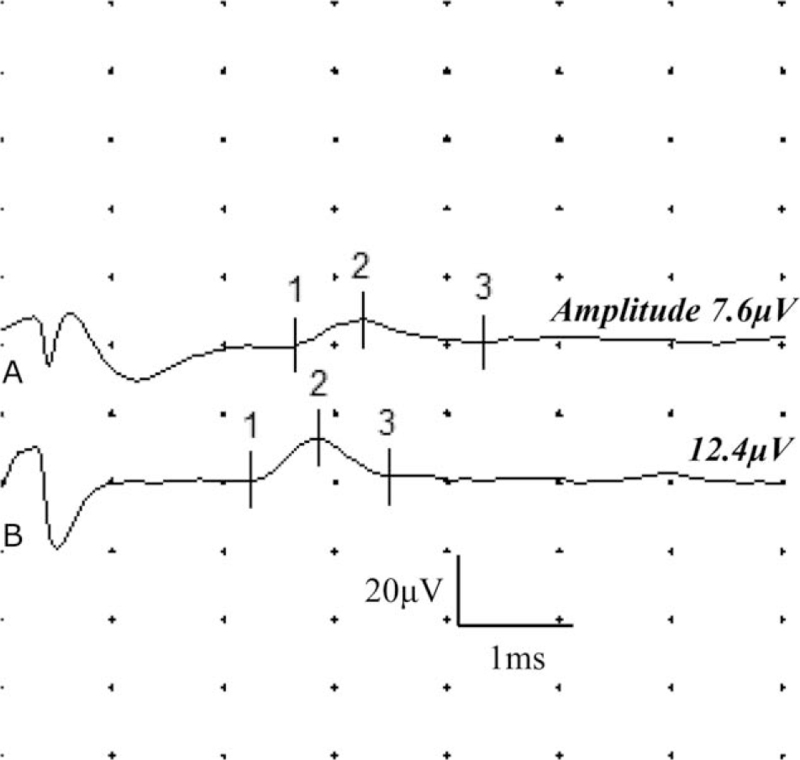
Results of the nerve conduction study of the left lateral dorsal cutaneous nerve at 1 month showed improvement in the delayed latency and decreased amplitude. (A) Initial; (B) follow-up at 1 month.

One month after the treatment, the pain in the patient's left ankle reduced to 3 points on the numeric rating scale. Moreover, causalgia and tingling sensation improved substantially, but allodynia persisted. In the follow-up electrodiagnostic test, partial recovery of amplitude and conduction velocity was confirmed in the sensory nerve conduction study of the left SN and LDCN (Fig. 3).

## 3 Discussion

CRPS is not a disease but a pathological exaggeration of the physiological responses resulting from misinterpretation and malprocessing of sensory information. Like many other medical syndromes, CRPS cannot be diagnosed using classical diagnostic techniques; hence, an accurate diagnosis only based on clinical signs and symptoms may be difficult. The main clue in CRPS diagnosis is history of previous injury and complaint of severe and persistent pain. Other typical features include hyperesthesia, vasomotor symptoms, hyperhydrosis, and trophic changes.^[11]^ The Budapest criteria are the accepted diagnostic criteria for CRPS (Table 2).^[12]^ According to the criteria, CRPS diagnosis requires the presence of at least one symptom in three or more of the following four categories: sensory, vasomotor, sudomotor/edema, and motor/trophic. Additionally, at least one objective test result in 2 or more of the above four categories must be confirmed. The symptoms presented in this case, including causalgia, allodynia, skin redness, swelling, and temperature asymmetry in the left ankle, belong to the sensory, vasomotor, and sudomotor/edema categories. Moreover, the electrodiagnostic study, three-phase bone scan, and digital infrared thermal imaging examination revealed abnormal findings. Finally, infection, vascular disorders, stress fractures, referred pain, and metabolic or inflammatory disorders were excluded, and a diagnosis of CRPS was made according to the Budapest diagnostic criteria.

LDCN injury is a rare injury that may result from a lateral ankle sprain or damage during surgeries or procedures performed for the Achilles tendon and lateral side of the ankle.^[7,8]^ The LDCN branches out as the SN passes under the lateral malleolus, and it is responsible for the sensory innervation of the dorsolateral aspect of the foot.^[5–7]^ SN conduction is important for diagnosing nerve damage or entrapment and is the most useful test for detecting electrophysiological abnormalities in neuropathy cases.^[7]^ Electrodiagnostic evaluation of the distal SN can be conveniently performed by a nerve conduction study, which can be useful in the differential diagnosis of LDCN injury.^[7,13]^

For the LDCN conduction study in this case, an active surface recording electrode was attached to the dorsolateral surface of the midpoint of the fifth metatarsal, and a bipolar stimulator was used in the inferoposterior portion of the lateral malleolus, 12 cm proximal from the recording electrode. The results showed delay in onset latency and a decrease in amplitude and conduction velocity compared to the contralateral side, thus indicating left LDCN injury. The onset latency, amplitude, and conduction velocity improved in the follow-up electrodiagnostic examination (Fig. 3).

CRPS is classified as type I or type II according to the absence or presence of nerve injury, respectively. Among the CRPS type II cases with proven nerve injury, carpal tunnel syndrome due to median nerve injury is most commonly reported.^[4,14]^ Several cases diagnosed as CRPS type II after nerve injury due to iatrogenic causes have been reported. In a study by Reynaldo et al, a case in which acute carpal tunnel syndrome and CRPS coexisted due to median nerve injury after radial artery cannulation was reported. A study by Park et al^[2]^ reported a CRPS type II case caused by cervical root injury after cervical transforaminal epidural injection.^[15]^ This is the first case of nerve injury of iatrogenic cause due to direct needle injury to the LDCN that progressed to CRPS type II. Various methods for treating CRPS have been reported, and active treatment in the early stages of CRPS is known to improve the prognosis. The treatment methods using physical therapy, corticosteroid administration, and transcutaneous nerve stimulation have been reported to be very successful.^[11]^ Corticosteroids are useful for reducing pain and swelling and improving mobility in CRPS patients.^[16]^ Furthermore, at an early stage in the treatment of CRPS, it is important to maintain the movement of the affected regions as much as possible; sensory stimulation is also recommended.^[17]^ Our treatment protocol aimed at reducing pain and paresthesia and restoring function. In this case, the patient showed rapid pain relief after early pharmacotherapy and comprehensive physical therapy; thus, a good prognosis is expected based on this progress.

In conclusion, LDCN injury should be considered in patients who complain of persistent lateral ankle and foot paresthesia or pain after sprain or procedures performed on the lateral side of the ankle. Early diagnosis and treatment can lead to good prognosis in cases where the LDCN injury has progressed to CRPS.

## Author contributions

**Conceptualization:** Hwan-Kwon Do.

**Formal analysis:** Wanil Kim.

**Investigation:** Tae-Hoon Kim, Geun-Yeol Jo, Hwan-Kwon Do.

**Supervision:** Geun-Yeol Jo, Wanil Kim.

**Writing – original draft:** Tae-Hoon Kim.

**Writing – review & editing:** Hwan-Kwon Do.
